# Trends in Cancer Mortality Among Black Individuals in the US From 1999 to 2019

**DOI:** 10.1001/jamaoncol.2022.1472

**Published:** 2022-05-19

**Authors:** Wayne R. Lawrence, Jennifer K. McGee-Avila, Jacqueline B. Vo, Qianlai Luo, Yingxi Chen, Maki Inoue-Choi, Amy Berrington de González, Neal D. Freedman, Meredith S. Shiels

**Affiliations:** 1 Division of Cancer Epidemiology and Genetics, National Cancer Institute, National Institutes of Health, Rockville, Maryland

## Abstract

**Importance:**

Cancer is the second leading cause of mortality in the US. Despite national decreases in cancer mortality, Black individuals continue to have the highest cancer death rates.

**Objective:**

To examine national trends in cancer mortality from 1999 to 2019 among Black individuals by demographic characteristics and to compare cancer death rates in 2019 among Black individuals with rates in other racial and ethnic groups.

**Design, Setting, and Participants:**

This serial cross-sectional study used US national death certificate data obtained from the National Center for Health Statistics and included all cancer deaths among individuals aged 20 years or older from January 1999 to December 2019. Data were analyzed from June 2021 to January 2022.

**Exposures:**

Age, sex, and race and ethnicity.

**Main Outcomes and Measures:**

Trends in age-standardized mortality rates and average annual percent change (AAPC) in rates were estimated by cancer type, age, sex, and race and ethnicity.

**Results:**

From 1999 to 2019, 1 361 663 million deaths from cancer occurred among Black individuals. The overall cancer death rate significantly decreased among Black men (AAPC, −2.6%; 95% CI, −2.6% to −2.6%) and women (AAPC, −1.5%; 95% CI, −1.7% to −1.3%). Death rates decreased for most cancer types, with the greatest decreases observed for lung cancer among men (AAPC, −3.8%; 95% CI, −4.0% to −3.6%) and stomach cancer among women (AAPC, −3.4%; 95% CI, −3.6% to −3.2%). Lung cancer mortality also had the largest absolute decreases among men (−78.5 per 100 000 population) and women (−19.5 per 100 000 population). We observed a significant increase in deaths from liver cancer among men (AAPC, 3.8%; 95% CI, 3.0%-4.6%) and women (AAPC, 1.8%; 95% CI, 1.2%-2.3%) aged 65 to 79 years. There was also an increasing trend in uterus cancer mortality among women aged 35 to 49 years (2.9%; 95% CI, 2.3% to 2.6%), 50 to 64 years (2.3%; 95% CI, 2.0% to 2.6%), and 65 to 79 years (1.6%; 95% CI, 1.2% to 2.0%). In 2019, Black men and women had the highest cancer mortality rates compared with non-Hispanic American Indian/Alaska Native, Asian or Pacific Islander, and White individuals and Hispanic/Latino individuals.

**Conclusions and Relevance:**

In this cross-sectional study, there were substantial decreases in cancer death rates among Black individuals from 1999 to 2019, but higher cancer death rates among Black men and women compared with other racial and ethnic groups persisted in 2019. Targeted interventions appear to be needed to eliminate social inequalities that contribute to Black individuals having higher cancer mortality.

## Key Points

**Questions:**

How did cancer mortality among Black individuals change in the US from 1999 to 2019 by age, sex, and cancer site, and how did 2019 cancer mortality rates among Black individuals compare with rates in other racial and ethnic groups?

**Findings:**

In this cross-sectional study of 1 361 663 deaths from cancer among Black individuals, although cancer mortality decreased considerably among Black individuals from 1999 to 2019, the cancer mortality rate was higher among Black men and women than in other racial and ethnic groups in 2019.

**Meaning:**

The findings suggest that resources should be allocated toward eliminating social inequalities and barriers throughout the cancer control continuum that contribute to substantially higher cancer mortality rates among Black men and women.

## Teaser

This cross-sectional study examines trends in cancer mortality among Black individuals in the US from 1999 to 2019 and compares mortality rates in 2019 between the Black population and other racial and ethnic populations.

## Introduction

Cancer is the second leading cause of mortality in the US.^1^ Despite annual decreases in cancer mortality, death rates among Black individuals remain higher than in other racial and ethnic groups.^2^ Detailed understanding of cancer mortality trends among Black individuals is essential to assess recent progress and to inform interventions aimed at addressing disparities. Therefore, we assessed trends in cancer mortality rates from 1999 to 2019 among Black adults by cancer site, age, and sex and compared cancer mortality rates in 2019 among Black men and women with rates in other racial and ethnic groups.

## Methods

For this cross-sectional study, demographic characteristics and causes of death were ascertained from national death certificate data from the National Center for Health Statistics from January 1999 to December 2019 (eTable 1 in the Supplement). Data were analyzed from June 2021 to January 2022. The National Institutes of Health institutional review board waived approval and informed consent because the study used publicly available deidentified data. This study followed the Strengthening the Reporting of Observational Studies in Epidemiology (STROBE) reporting guideline.

Our main analysis focused on age-adjusted cancer death rates by age group, sex, and cancer site among non-Hispanic Black individuals aged 20 years or older. All rates were age-standardized in 5-year age groups to the 2000 US population. Joinpoint regression was used to estimate average annual percent changes (AAPCs) in mortality rates and APCs to identify calendar years during which significant changes in trajectories occurred and to assess the trend in each segment. Age-standardized cancer death rates in 2019 were used to compare cancer death rates between Black individuals and Hispanic/Latino and non-Hispanic American Indian/Alaska Native, Asian or Pacific Islander, and White individuals. American Indian/Alaska Native death rates were restricted to Indian Health Service Purchased/Referred Care Delivery Areas to increase ascertainment of this group on death certificates. SEER*Stat software, version 8.3.9 was used to estimate age-adjusted mortality rates.

## Results

From 1999 to 2019, 1 361 663 million cancer deaths occurred among Black individuals aged 20 years or older. The age-adjusted death rate was 377.3 per 100 000 population among Black men and 239.4 per 100 000 population among Black women (eTable 2 in the Supplement).

**Figure 1.**
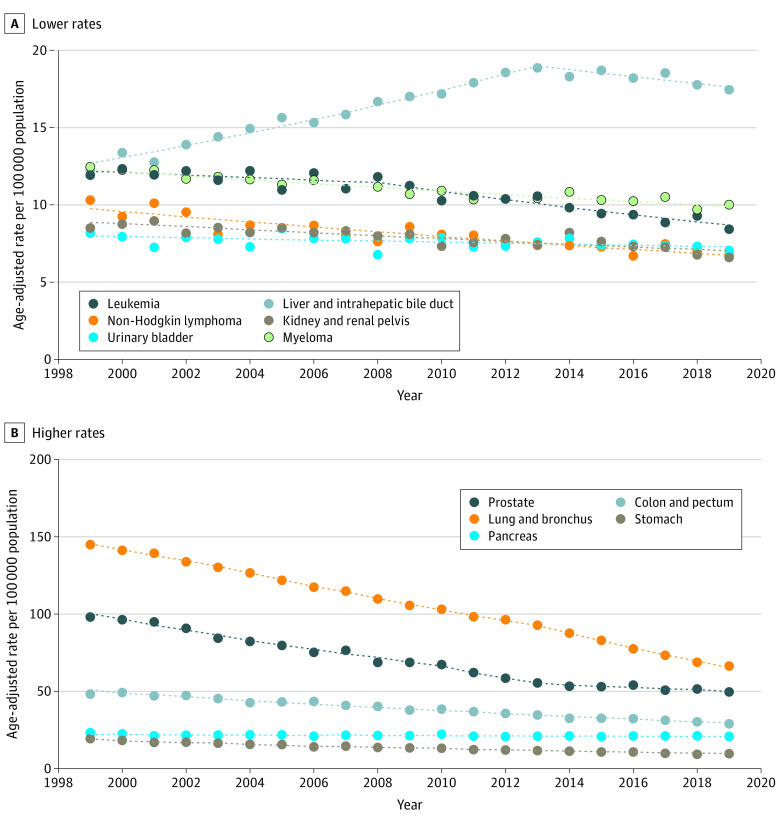
Trends in Age-Standardized Death Rates Among Black Men by Cancer Site From 1999 to 2019 Death rates are calculated from the entire US population. Trends were estimated using joinpoint regression. Filled circles represent observed age-adjusted rates; dotted lines, modeled age-adjusted rates.

**Figure 2.**
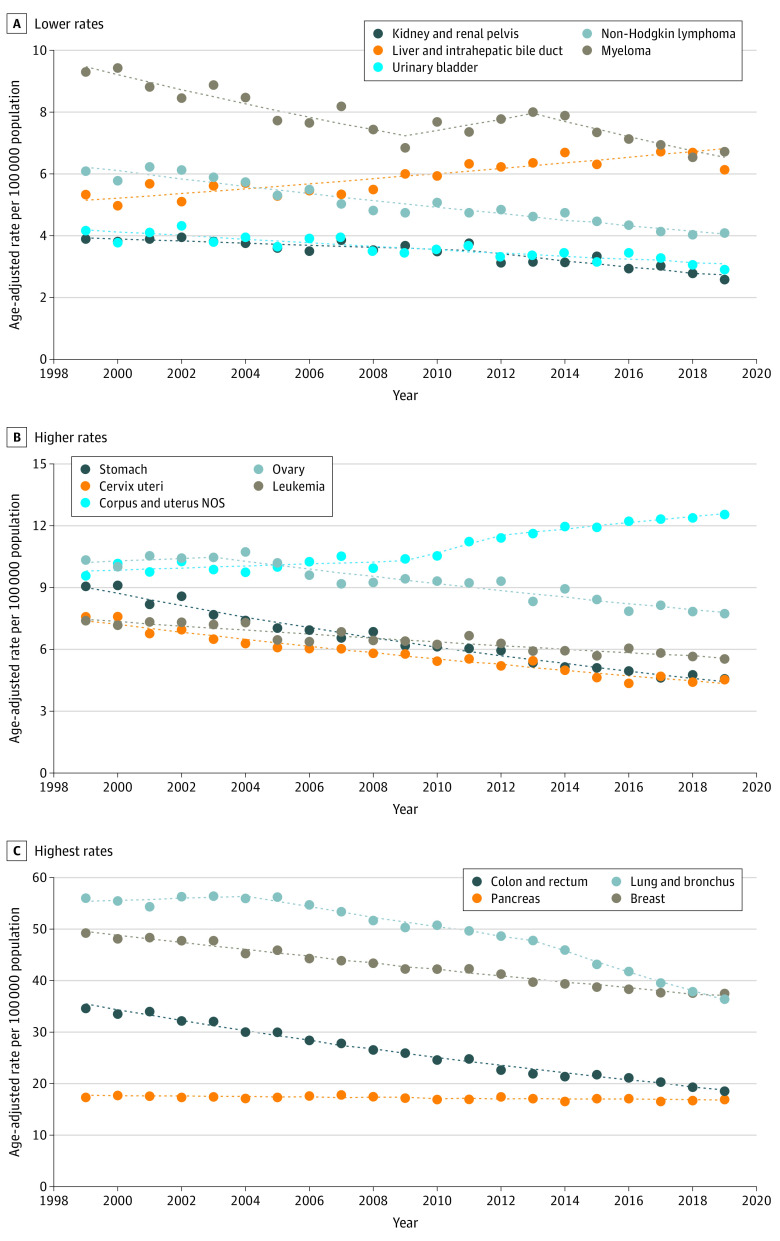
Trends in Age-Standardized Death Rates Among Black Women by Cancer Site From 1999 to 2019 Death rates are calculated from the entire US population. Trends were estimated using joinpoint regression. Filled circles represent observed age-adjusted rates; dotted lines, modeled age-adjusted rates. NOS indicates not otherwise specified.

Cancer mortality rates among Black individuals decreased 2.0% per year from 1999 to 2019 (change, −120.1 per 100 000 population), with a larger reduction among men (change, −200.1 per 100 000 population; AAPC, −2.6% [95% CI, −2.6% to −2.6%]) than among women (−74.8 per 100 000 population; AAPC −1.5% [95% CI, −1.7% to −1.3%]) (eTable 3 in the Supplement). There were decreases in rates for most cancer sites among men except the liver, for which the rate increased from 1999 to 2013 (APC, 3.0%; 95% CI, 2.7%-3.3%) and later decreased (APC, −1.3%; 95% CI, −2.1% to −0.4%) (Figure 1). Similarly, among women, there were decreases in rates for most cancer sites except the liver (AAPC, 1.1%; 95% CI, 0.0%-2.3%) and uterus (AAPC, 1.3%; 95% CI, 0.5%-2.2%) (Figure 2). The greatest decreases were observed for lung cancer among men (AAPC, −3.8%; 95% CI, −4.0% to −3.6%) and stomach cancer among women (AAPC, −3.4%; 95% CI, −3.6% to −3.2%). Lung cancer mortality had the largest absolute decreases among men (−78.5 per 100 000 population) and women (−19.5 per 100 000 population).

Cancer death rates decreased in all age groups from 1999 to 2019 for most cancer sites among Black men and women (eFigures 1-3 in the Supplement). For men, the greatest decreases were for lung cancer among those aged 35 to 49 years, 50 to 64 years, and 65 to 79 years. Liver cancer mortality rates increased among men aged 65 to 79 years (AAPC, 3.8%; 95% CI, 3.0%-4.6%). For women, uterus cancer mortality increased among those aged 35 to 49 years (AAPC, 2.9%; 95% CI, 2.3%-3.6%), 50 to 64 years (AAPC, 2.3%; 95% CI, 2.0%-2.6%), and 65 to 79 years (AAPC, 1.6%; 95% CI, 1.2%-2.0%) and liver cancer mortality increased among those aged 65 to 79 years (AAPC, 1.8; 95% CI, 1.2%-2.3%).

In 2019, compared with other racial and ethnic groups, Black men and women had a higher cancer mortality rate overall and for most cancer sites (Table). Among men, the most pronounced difference was observed for deaths from prostate cancer; for example, rates among Black men were approximately 5-times higher than those among Asian or Pacific Islander men (51.3 per 100 000 population [95% CI, 49.8-52.8 per 100 000 population] vs 11.0 per 100 000 population [95% CI, 10.2-11.9 per 100 000 population]). Breast cancer mortality rates were nearly 2.5-times higher among Black women than among Asian or Pacific Islander women (39.0 per 100 000 population [95% CI, 38.0-39.9 per 100 000 population] vs 16.1% per 100 000 population [95% CI, 15.3-17.0 per 100 000 population]). Moreover, myeloma mortality rates were substantially higher among Black individuals than among Asian or Pacific Islander and American Indian/Alaska Native individuals.

**Table.** Age-Standardized Death Rates for the Most Common Causes of Death From Cancer by Sex and Racial and Ethnic Group in the US in 2019

Cancer site or type	Age-standardized death rate, per 100 000 population (95% CI)^a^				
	Non-Hispanic individuals				Hispanic/Latino individuals
	American Indian/Alaska Native^b^	Asian or Pacific Islander	Black	White	
**All sites**					
Men	255.2 (240.4-270.6)	149.5 (146.5-152.6)	294.1 (290.9-297.4)	249 (248.0-250.0)	176.7 (174.2-179.2)
Women	188.5 (177.5-199.9)	113.2 (110.9-115.5)	205.1 (202.9-207.3)	181.8 (181.0-182.6)	127.9 (126.1-129.7)
**Men**					
Lung and bronchus	52.0 (45.4-59.1)	33.6 (32.2-35.1)	68.6 (67.0-70.2)	59.5 (59.0-60.0)	28.7 (27.7-29.8)
Prostate	29.7 (24.3-35.9)	11.0 (10.2-11.9)	51.3 (49.8-52.8)	24.5 (24.2-24.8)	20.9 (20.0-21.8)
Colon and rectum	25.9 (21.4-31.0)	15.2 (14.3-16.2)	30.1 (29.1-31.1)	21.2 (20.9-21.5)	18.1 (17.4-18.9)
Pancreas	14.1 (10.9-17.9)	11.6 (10.8-12.5)	21.4 (20.6-22.3)	18.2 (17.9-18.4)	13.8 (13.2-14.5)
Liver and intrahepatic bile duct	23.4 (19.3-28.2)	17.3 (16.27-18.3)	17.9 (17.2-18.7)	11.8 (11.6-12.0)	17.9 (17.2-18.7)
Myeloma	4.7 (2.9-7.2)	2.5 (2.1-3.0)	10.3 (9.7-11.0)	5.2 (5.1-5.3)	4.3 (3.9-4.7)
Stomach	11.3 (8.5-14.8)	7.7 (7.0-8.5)	9.6 (9.01-10.2)	4.0 (3.9-4.1)	8.0 (7.5-8.6)
Leukemia	6.9 (4.7-9.7)	6.2 (5.5-6.8)	8.7 (8.1-9.3)	11.6 (11.3-11.8)	6.7 (6.3-7.2)
Urinary bladder	5.3 (3.3-8.1)	4.0 (3.5-4.5)	7.2 (6.7-7.8)	10.9 (10.7-11.1)	5.5 (5.1-6.0)
Non-Hodgkin lymphoma	7.9 (5.6-10.8)	6.8 (6.2-7.5)	6.9 (6.4-7.4)	9.6 (9.4-9.8)	7.7 (7.2-8.2)
Kidney and renal pelvis	11.0 (8.1-14.6)	3.2 (2.7-3.6)	6.8 (6.4-7.3)	7.3 (7.2-7.5)	6.5 (6.0-6.9)
**Women**					
Breast	27.3 (23.2-31.9)	16.1 (15.3-17.0)	39.0 (38.0-39.9)	27.1 (26.8-27.4)	19.5 (18.8-20.2)
Lung and bronchus	42.6 (37.5-48.1)	21.5 (20.5-22.5)	37.5 (36.6-38.5)	44.1 (43.8-44.5)	15.3 (14.6-15.9)
Colon and rectum	19.3 (15.9-23.2)	10.7 (10.0-11.4)	19.1 (18.4-19.8)	15.2 (15.0-15.5)	11.6 (11.1-12.2)
Pancreas	11.9 (9.3-15.1)	9.5 (8.8-10.2)	17.4 (16.7-18.0)	13.5 (13.3-13.8)	11.1 (10.6-11.6)
Corpus and uterus, NOS	7.6 (5.6-10.2)	5.1 (4.6-5.6)	12.9 (12.4-13.5)	6.5 (6.3-6.6)	6.0 (5.6-6.4)
Ovary	6.8 (4.9-9.3)	5.9 (5.4-6.5)	8.0 (7.5-8.4)	8.9 (8.7-9.0)	6.7 (6.3-7.2)
Myeloma	1.8 (0.9-3.3)	1.6 (1.4-1.9)	7.0 (6.6-7.4)	3.0 (2.9-3.1)	2.9 (2.7-3.2)
Liver and intrahepatic bile duct	12.0 (9.4-15.1)	7.1 (6.5-7.7)	6.3 (5.9-6.7)	5.1 (5.0-5.2)	8.1 (7.7-8.6)
Leukemia	4.8 (3.2-7.0)	3.4 (3.0-3.8)	5.7 (5.4-6.1)	6.2 (6.1-6.4)	4.5 (4.2-4.8)
Cervix uteri	4.4 (2.9-6.4)	2.1 (1.8-2.4)	4.7 (4.4-5.1)	2.8 (2.7-2.9)	3.3 (3.0-3.6)
Stomach	5.7 (4.0-8.1)	4.8 (4.4-5.3)	4.7 (4.3-5.0)	2.0 (2.0-2.1)	5.4 (5.0-5.7)
Non-Hodgkin lymphoma	4.9 (3.2-7.1)	3.6 (3.2-4.1)	4.2 (3.9-4.6)	5.7 (5.6-5.8)	4.6 (4.3-5.0)
Urinary bladder	1.6 (0.7-2.9)	1.4 (1.1-1.6)	3.0 (2.7-3.3)	3.1 (3.0-3.2)	1.7 (1.5-2.0)
Kidney and renal pelvis	5.3 (3.6-7.6)	1.3 (1.1-1.6)	2.7 (2.5-3.0)	3.0 (2.9-3.1)	2.9 (2.7-3.2)

Abbreviation: NOS, not otherwise specified.

^a^ All were age-adjusted to the 2000 US standard population.

^b^ Data for the non-Hispanic American Indian/Alaska Native population are restricted to Indian Health Service Purchased/Referred Care Delivery Area counties.

## Discussion

From 1999 to 2019, there were substantial decreases in cancer mortality rates among Black men and women in the US in all age groups studied. Decreases were observed for most cancer sites except the liver and uterus among older adults, and the greatest decreases were observed for lung cancer among men and stomach cancer among women. Despite decreases in cancer mortality among Black individuals, cancer mortality rates in 2019 were higher in this group than in other racial and ethnic groups included in the analysis.

Decreases in cigarette smoking and improvements in early detection and targeted cancer treatment plans have been shown to be associated with decreases in cancer mortality among Black men and women.^3^ In the US, smoking prevalence among Black individuals decreased from 24.3% in 1999 to 14.9% in 2019, contributing to decreases in deaths from lung, bladder, kidney, and pancreatic cancer.^1,4,5,6^ Decreases in breast cancer mortality among Black women may be associated with increased accessibility to screening, earlier detection, and advances in treatment.^7,8^ In addition, decreases in colorectal cancer mortality, particularly among Black individuals aged 50 years or older, may be associated with increased access to both invasive and noninvasive screening.^7^ Decreasing rates of death from prostate cancer may be associated with improved treatment and hormonal therapy for advanced disease.^9^ However, the slowing trend in prostate cancer mortality rates among older men might be attributable to the increasing incidence of distant stage disease, which may be associated with the 2012 US Preventive Task Force recommendation against prostate-specific antigen screening.^10^

Trends in liver cancer mortality rates among Black individuals differed by sex and age group. Increasing mortality rates in older age groups may be associated with these groups having the highest national prevalence of chronic hepatitis C virus infection and a higher prevalence of obesity, alcohol use, and metabolic disorders.^6,11,12^ The reduction in liver cancer mortality in recent years in some age groups may be associated with improvements in screening for hepatitis C virus infection and treatment for hepatitis B and C virus infection as well as with liver dysfunction in individuals with liver cancer.^12^ After accounting for hysterectomy, increasing uterine cancer death rates among Black women have been shown to be associated with longer duration of excess adiposity, largely owing to greater exposure to stressors and neighborhood-level factors.^6,7^

Despite national progress in reducing cancer mortality, Black individuals had considerably higher cancer mortality rates in 2019 compared with other racial and ethnic groups. The factors associated with racial disparities in cancer death rates are primarily systemic and preventable. Black patients are more likely to experience poor patient-physician interactions, longer referral times, delays in treatment, greater medical mistrust, underuse of treatment, and health care system failure—all mutable factors.^8,13,14^ In addition, Black individuals are more likely to reside in neighborhoods with poor accessibility to specialists, see physicians with fewer clinical resources, and live in communities with greater exposure to environmental toxins.^8^ Therefore, examining individual-level behavioral and biological factors is insufficient, and greater emphasis should be aimed at understanding the contribution of social inequities to higher cancer mortality rates among Black individuals. Addressing inequalities that contribute to racial disparities in cancer mortality requires policies that seek to resolve adverse socioenvironmental conditions and determinants that contribute to inequities throughout the entire continuum of care.

### Limitations

The main limitations of this study include potential misclassification of cause of death and race and ethnicity on death certificates and the use of broad groupings of race and ethnicity that can mask differences within groups. Moreover, decreases in cancer death rates may slow in future years in association with the COVID-19 pandemic, which has disproportionately affected Black communities in the US.^15^

## Conclusions

In this cross-sectional study, there were substantial decreases in cancer death rates among Black individuals from 1999 to 2019. This decrease may have been associated with advances in cancer prevention, detection, and treatment as well as with population changes in exposure to cancer risk factors. However, in 2019, Black individuals continued to have the highest cancer mortality rates compared with other racial and ethnic groups, suggesting a need to address the pervasiveness of longstanding racial and ethnic inequities. Eliminating racial and ethnic disparities in cancer mortality will require equitable access to cancer prevention, early diagnosis, and timely and guideline-adherent high-quality care.

## Notes

Supplement. **eTable 1.** Cancer Mortality by Site Based on *ICD-10* Codes **eTable 2.** Age-Adjusted Mortality Rates for the Most Common Causes of Cancer Death by Sex Among Black Individuals in the United States, 1999-2019 **eTable 3.** Annual Percentage Changes in Cancer Death Rates by Sex and Cancer Site Among Black Individuals From 1999 to 2019 in the United States **eFigure 1.** Trends in Age-standardized Cancer Death Rates (1999-2019) Among Black Individuals by Age Group **eFigure 2.** Trends in Age-standardized Death Rates (1999-2019) Among Black Men by Cancer Site and Age Group **eFigure 3.** Trends in Age-standardized Death Rates (1999-2019) Among Black Women by Cancer Site and Age Group
