# Supplementary material for: Trends in Cancer Mortality Among Black Individuals in the US From 1999 to 2019
Source: JAMA Oncol. 2022 May 19;8(8):1184–9. doi: 10.1001/jamaoncol.2022.1472 (PMC9121301; doi:10.1001/jamaoncol.2022.1472)
Supplement: Supplement. — eTable 1. Cancer Mortality by Site Based on ICD-10 Codes eTable 2. Age-Adjusted Mortality Rates for the Most Common Causes of Cancer Death by Sex Among Black Individuals in the United States, 1999-2019 eTable 3. Annual Percentage Changes in Cancer Death Rates by Sex and Cancer Site Among Black Individuals From 1999 to 2019 in the United States eFigure 1. Trends in Age-standardized Cancer Death Rates (1999-2019) Among Black Individuals by Age Group eFigure 2. Trends in Age-standardized Death Rates (1999-2019) Among Black Men by Cancer Site and Age Group eFigure 3. Trends in Age-standardized Death Rates (1999-2019) Among Black Women by Cancer Site and Age Group [file jamaoncol-e221472-s001.pdf]

Supplemental Online Content

Lawrence WR, McGee-Avila JK, Vo JB, et al. Trends in cancer mortality among Black individuals in the US from 1999 to 2019. *JAMA Oncol*. Published online May 19, 2022. doi:10.1001/jamaoncol.2022.1472

eTable 1. Cancer Mortality by Site Based on *ICD-10* Codes

eTable 2. Age-Adjusted Mortality Rates for the Most Common Causes of Cancer Death by Sex Among Black Individuals in the United States, 1999-2019

eTable 3. Annual Percentage Changes in Cancer Death Rates by Sex and Cancer Site Among Black Individuals From 1999 to 2019 in the United States

eFigure 1. Trends in Age-standardized Cancer Death Rates (1999-2019) Among Black Individuals by Age Group

eFigure 2. Trends in Age-standardized Death Rates (1999-2019) Among Black Men by Cancer Site and Age Group

eFigure 3. Trends in Age-standardized Death Rates (1999-2019) Among Black Women by Cancer Site and Age Group

This supplemental material has been provided by the authors to give readers additional information about their work.

© 2022 Lawrence WR et al. *JAMA Oncology.*

**eTable 1.** Cancer mortality by site based on ICD-10 codes	
SEER Cause of Death Label	ICD-10
All Malignant Cancers	C00-C97
Breast	C50
Cervix Uteri	C53
Colon and Rectum	C18, C20
Corpus and Uterus, NOS	C54-C55
Kidney and Renal Pelvis	C64-C65
Leukemia	C91-C95
Liver and Intrahepatic Bile	C22
Lung and Bronchus	C34
Non-Hodgkin Lymphoma	C82, C83, C85
Ovary	C56
Pancreas	C25
Prostate	C61
Stomach	C16
Urinary Bladder	C67
Abbreviations: ICD-10, International Statistical Classification of Diseases and Related Health Problems, Tenth Revision	

© 2022 Lawrence WR et al. *JAMA Oncology.*

**eTable 2.** Age-adjusted mortality rates for the most common causes of cancer death by sex among Black individuals in the United States, 1999-2019			
		1999-2019 (Per 100,000)	
cancer site or type		Men	Women
All malignant cancers		377.3	239.4
Lung and Bronchus		104.2	49.9
Prostate		68.5	NA
Female Breast		NA	43.6
Colon and Rectum		38.7	26.1
Pancreas		21.8	17.6
Ovary		NA	9.4
Leukemia		10.8	6.5
Corpus and Uterus, NOS		NA	11.3
Liver and intrahepatic bile duct		17.4	6.2
Non-Hodgkin Lymphoma		8.2	5.1
Urinary Bladder		7.7	3.6
Kidney and Renal Pelvis		8.0	3.5
Stomach		13.4	6.4
Myeloma		11.1	7.9
Cervix Uteri		NA	5.8

Rates are per 100,000 and age-adjusted to the 2000 US Std Population (19 age groups - Census P25-1130) standard.
Abbreviation: NOS = not otherwise specified; NA, not applicable

© 2022 Lawrence WR et al. *JAMA Oncology.*

eTable 3. Annual percentage changes in cancer death rates by sex and cancer site among Black individuals from 1999 to 2019 in the United States									
Cause of death	Average APC from (1999-2019)	Segment 1		Segment 2		Segment 3		Segment 4	
**All Malignant Cancers**									
	-2.0 (-2.1, -2.0)	1999-2019	-2.0 (-2.1, -2.0)	NA	NA	NA	NA	NA	NA
**Men**									
Overall cancer	-2.6 (-2.6, -2.6)	1999-2019	-2.6 (-2.6, -2.6)	NA	NA	NA	NA	NA	NA
Prostate	-3.4 (-3.8, -3.0)	1999-2014	-4.0 (-4.3, -3.7)	2014-2019	-1.6 (-3.3, 0.0)	NA	NA	NA	NA
Colon and Rectum	-2.6 (-2.7, -2.4)	1999-2019	-2.6 (-2.7, -2.4)	NA	NA	NA	NA	NA	NA
Kidney and Renal Pelvis	-1.1 (-1.4, -0.8)	1999-2019	-1.1 (-1.4, -0.8)	NA	NA	NA	NA	NA	NA
Pancreas	-0.2 (-0.4, -0.1)	1999-2019	-0.2 (-0.4, -0.1)	NA	NA	NA	NA	NA	NA
Lung and Bronchus	-3.8 (-4.0, -3.6)	1999-2003	-2.5 (-3.2, -1.8)	2003-2013	-3.3 (-3.5, -3.1)	2013-2019	-5.5 (-5.9, -5.1)	NA	NA
Liver and Intrahepatic Bile	1.7 (1.4, 2.0)	1999-2013	3.0 (2.7, 3.3)	2013-2019	-1.3 (-2.1, -0.4)	NA	NA	NA	NA
Urinary Bladder	-0.4 (-0.7, -0.0)	1999-2019	-0.4 (-0.7, -0.0)	NA	NA	NA	NA	NA	NA
Stomach	-3.2 (-3.4, -3.0)	1999-2019	-3.2 (-3.4, -3.0)	NA	NA	NA	NA	NA	NA
Leukemia	-1.6 (-2.1, -1.1)	1999-2008	-0.6 (-1.6, 0.3)	2008-2019	-2.4 (-3.1, -1.8)	NA	NA	NA	NA
Non-Hodgkin Lymphoma	-1.8 (-2.1, -1.4)	1999-2019	-1.8 (-2.1, -1.4)	NA	NA	NA	NA	NA	NA
Myeloma	-1.0 (-1.2, -0.8)	1999-2019	-1.0 (-1.2, -0.8)	NA	NA	NA	NA	NA	NA
**Women**									
Overall cancer	-1.5 (-1.7, -1.3)	1999-2002	-0.9 (-2.1, 0.2)	2002-2019	-1.6 (-1.7, -1.5)	NA	NA	NA	NA
Breast	-1.4 (-1.5, -1.3)	1999-2019	-1.4 (-1.5, -1.3)	NA	NA	NA	NA	NA	NA
Colon and Rectum	-3.0 (-3.2, -2.9)	1999-2019	-3.0 (-3.2, -2.9)	NA	NA	NA	NA	NA	NA
Kidney and Renal Pelvis	-1.8 (-2.4, -1.1)	1999-2011	-0.8 (-1.6, -0.1)	2011-2019	-3.1 (-4.5, -1.8)	NA	NA	NA	NA
Pancreas	-0.2 (-0.3, -0.1)	1999-2019	-0.2 (-0.3, -0.1)	NA	NA	NA	NA	NA	NA
Lung and Bronchus	-2.0 (-2.3, -1.8)	1999-2005	0.1 (-0.5, 0.7)	2005-2013	-1.9 (-2.3, -1.4)	2013-2019	-4.4 (-4.9, -3.8)	NA	NA

© 2022 Lawrence WR et al. *JAMA Oncology.*

Liver and Intrahepatic Bile	1.1 (0.0, 2.3)	1999-2017	1.8 (1.3, 2.2)	2017-2019	-4.4 (-14.7, 7.2)	NA	NA	NA	NA
Urinary Bladder	-1.4 (-1.8, -1.1)	1999-2019	-1.4 (-1.8, -1.1)	NA	NA	NA	NA	NA	NA
Stomach	-3.4 (-3.6, -3.2)	1999-2019	-3.4 (-3.6, -3.2)	NA	NA	NA	NA	NA	NA
Ovary	-1.3 (-2.9, -0.4)	1999-2004	1.0 (-0.9, 3.0)	2004-2007	-4.7 (-12.5, 3.8)	2007-2010	0.9 (-7.3, 9.9)	2010-2019	-2.2 (-2.9, -1.4)
Cervix Uteri	-2.5 (-2.8, -2.2)	1999-2019	-2.5 (-2.8, -2.2)	NA	NA	NA	NA	NA	NA
Corpus and Uterus, NOS	1.3 (0.5, 2.2)	1999-2009	0.6 (-0.1, 1.1)	2009-2012	4.0 (-1.9, 10.4)	2012-2019	1.3 (0.6, 2.0)	NA	NA
Leukemia	-1.4 (-1.6, -1.1)	1999-2019	-1.4 (-1.6, -1.1)	NA	NA	NA	NA	NA	NA
Non-Hodgkin Lymphoma	-2.0 (-2.3, -1.8)	1999-2019	-2.0 (-2.3, -1.8)	NA	NA	NA	NA	NA	NA
Myeloma	-1.7 (-2.8, -0.7)	1999-2009	-2.6 (-3.4, -1.8)	2009-2013	2.5 (-2.6, 7.9)	2013-2019	-3.1 (-4.7, -1.5)	NA	NA

Segments were chosen by Joinpoint regression.
Abbreviations: APC, Annual percentage change; NA, not applicable

© 2022 Lawrence WR et al. *JAMA Oncology.*

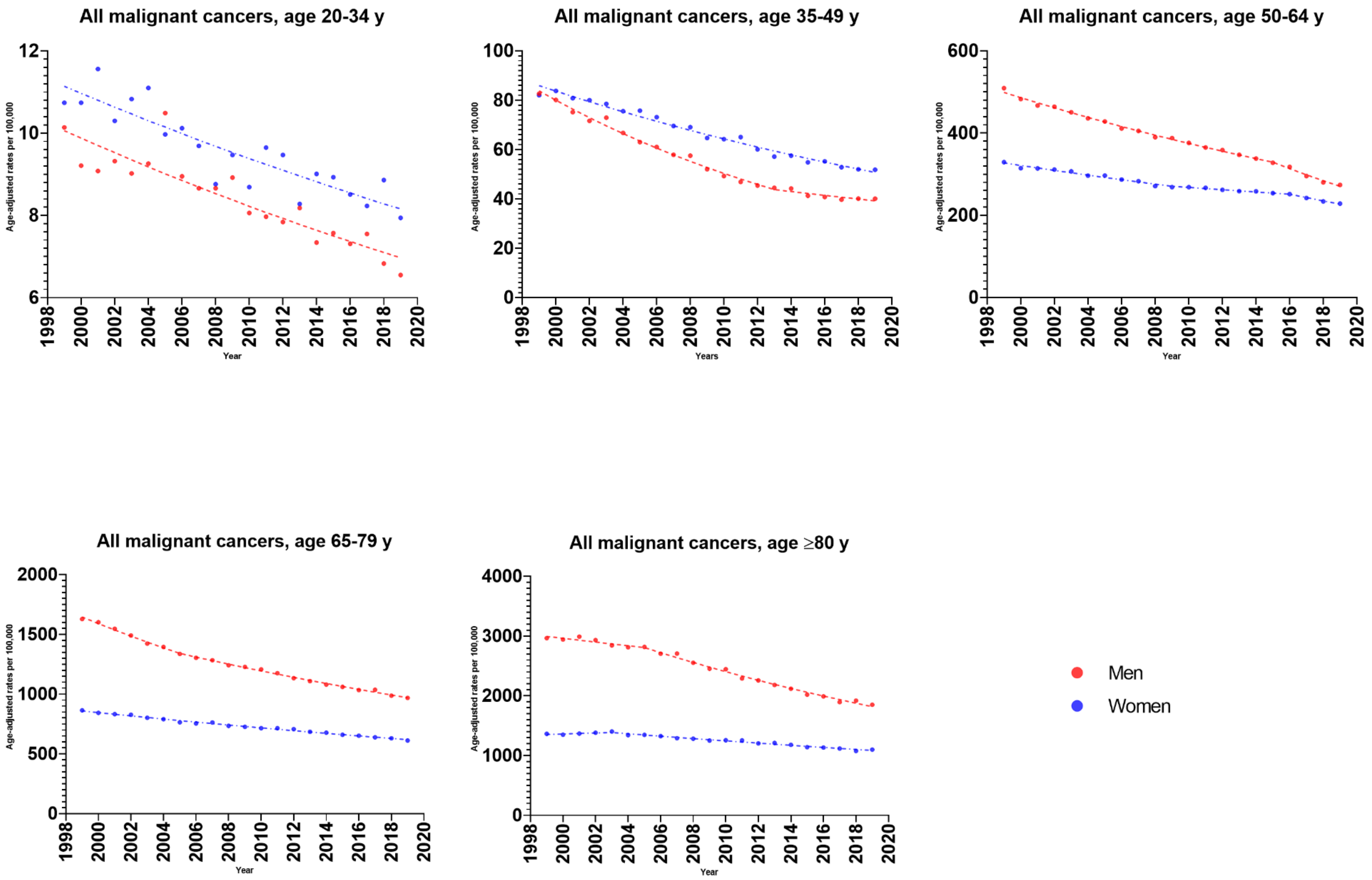

eFigure 1. Trends in age-standardized cancer death rates (1999-2019) among Black individuals by age group. Death rates covered the entire US population. Trends were estimated using joinpoint regression and characterized by rate per 100,000.
Note: "---"represents modeled age-adjusted and "●" represents observed age-adjusted rates.

© 2022 Lawrence WR et al. *JAMA Oncology.*

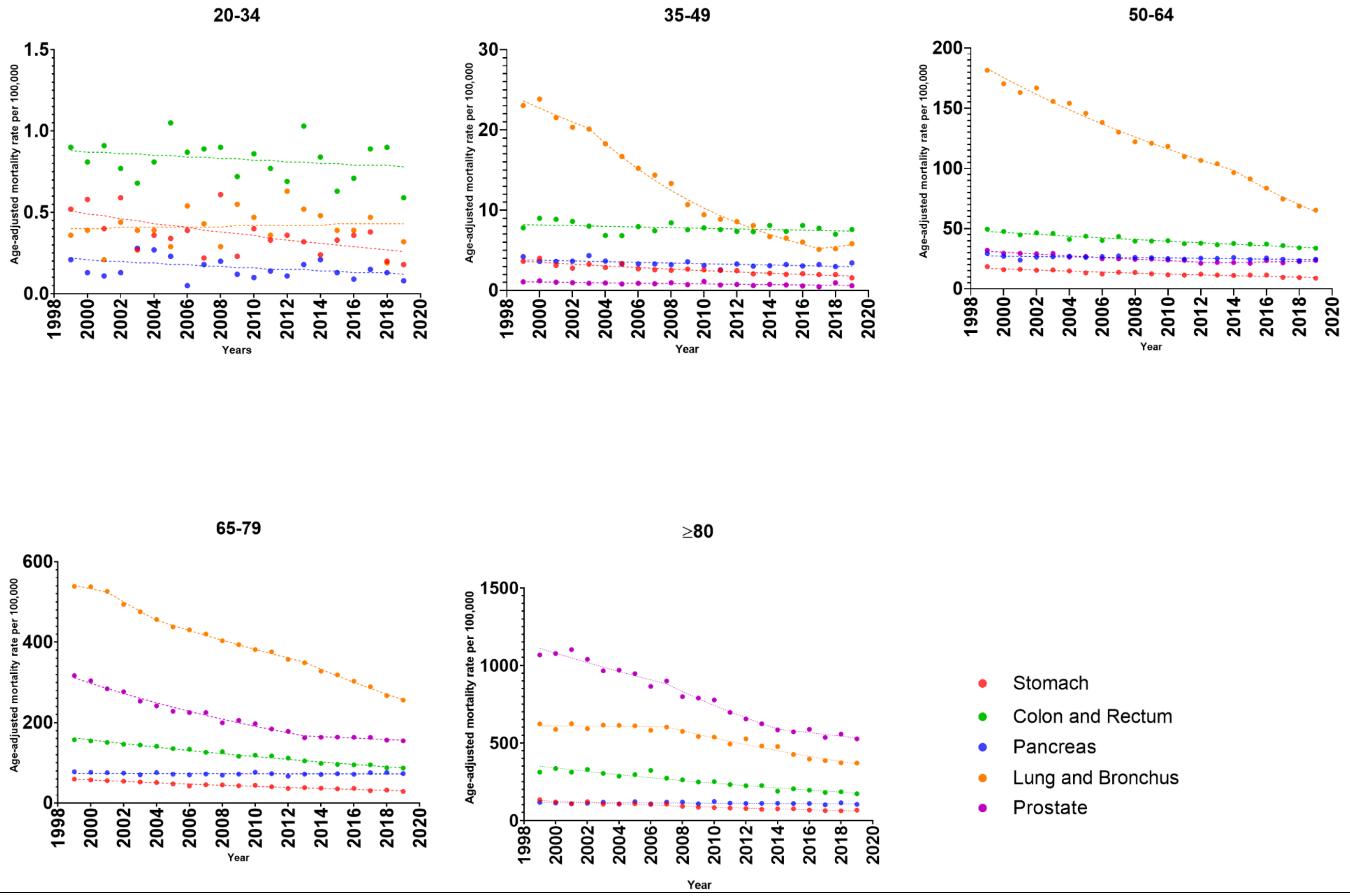

eFigure 2. Trends in age-standardized death rates (1999-2019) among Black men by cancer site and age group. Death rates covered the entire US population. Trends were estimated using joinpoint regression and characterized by rate per 100,000.
Note: “---“represents modeled age-adjusted and “●” represents observed age-adjusted rates.

© 2022 Lawrence WR et al. *JAMA Oncology.*

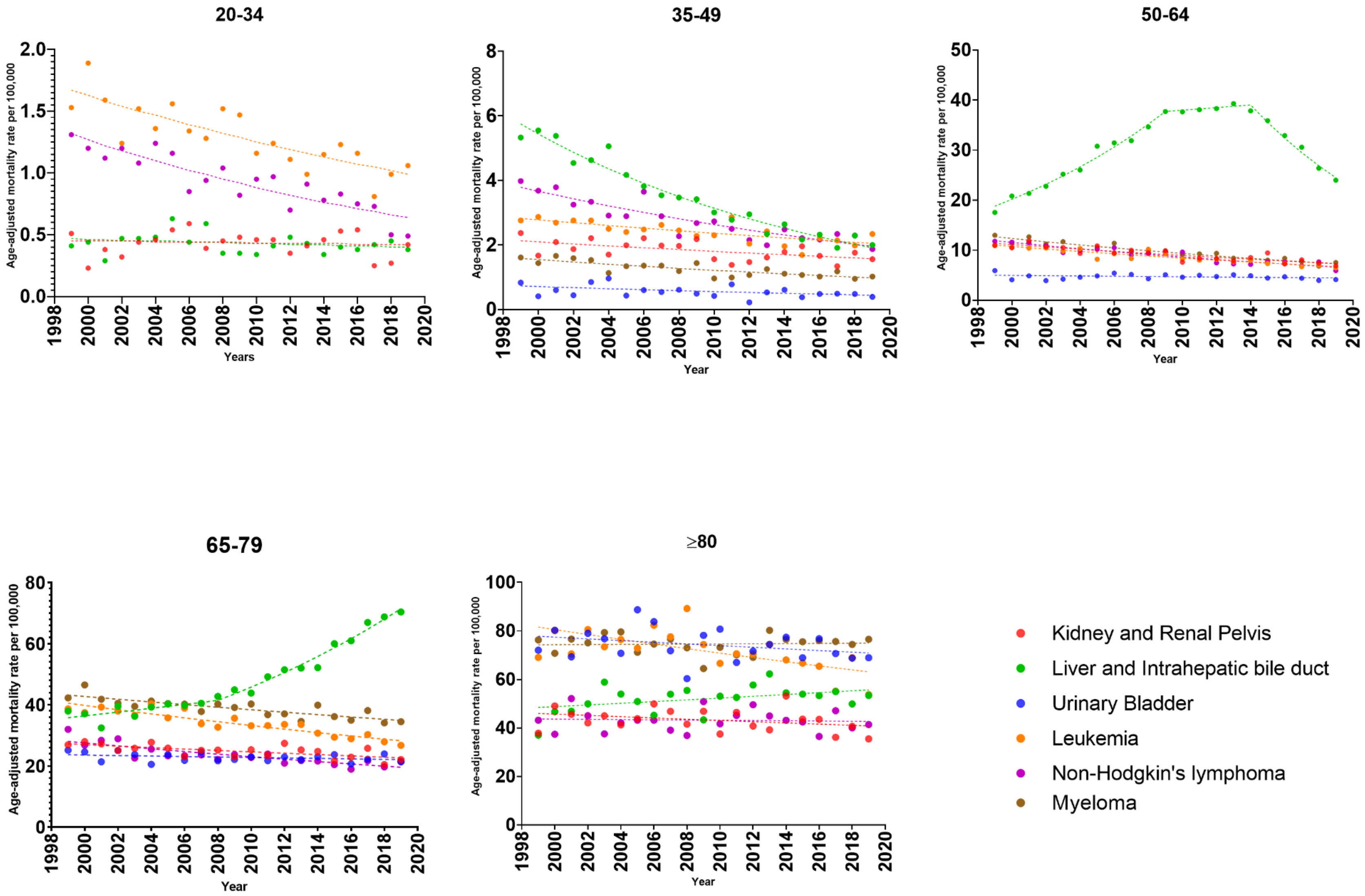

(Continued) eFigure 2. Trends in age-standardized death rates (1999-2019) among Black men by cancer site and age group. Death rates covered the entire US population. Trends were estimated using joinpoint regression and characterized by rate per 100,000.
Note: "---"represents modeled age-adjusted and "●" represents observed age-adjusted rates.

© 2022 Lawrence WR et al. *JAMA Oncology.*

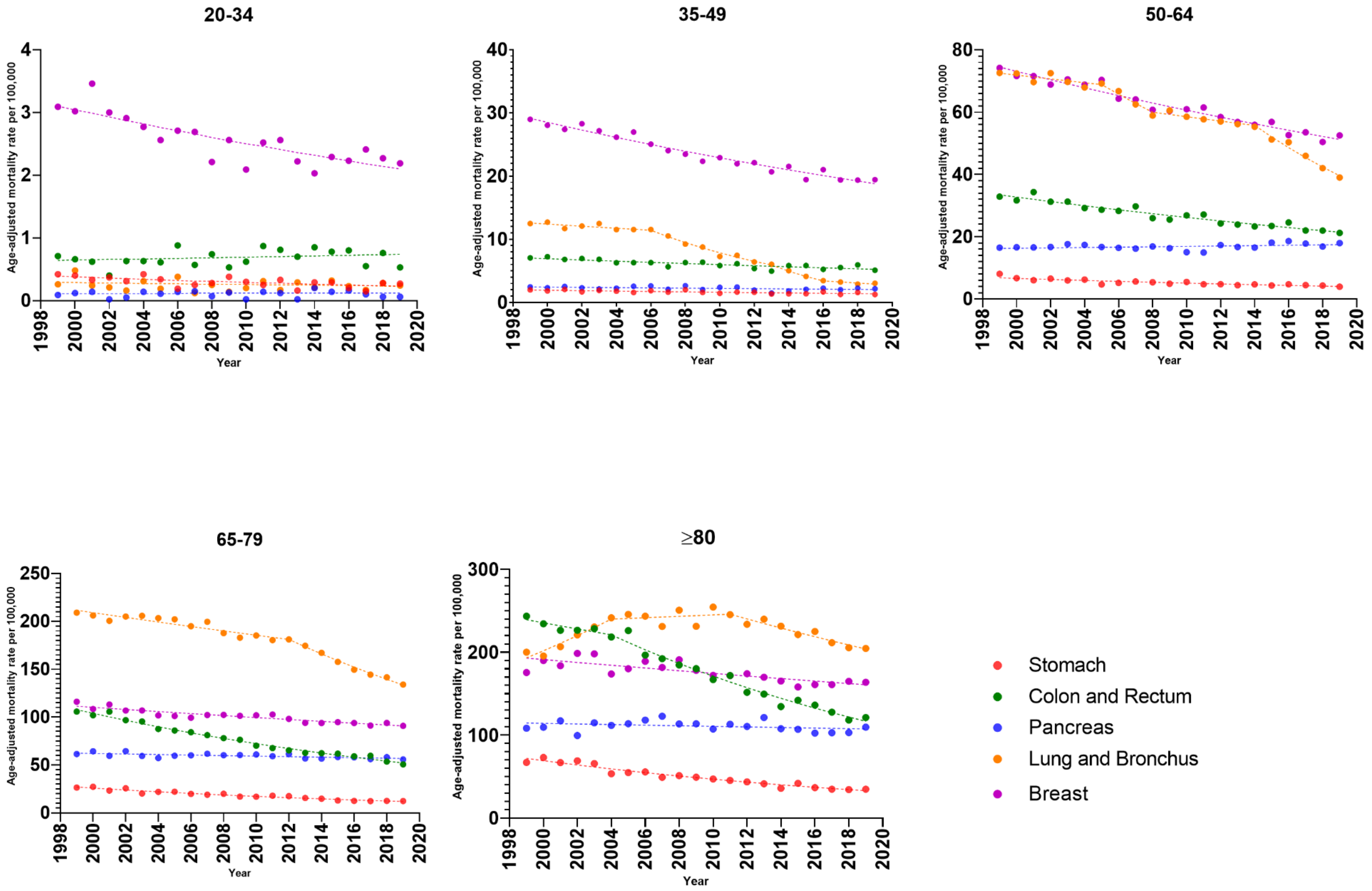

eFigure 3. Trends in age-standardized death rates (1999-2019) among Black women by cancer site and age group. Death rates covered the entire US population. Trends were estimated using joinpoint regression and characterized by rate per 100,000.
Note: “---“represents modeled age-adjusted and “●” represents observed age-adjusted rates

© 2022 Lawrence WR et al. *JAMA Oncology.*

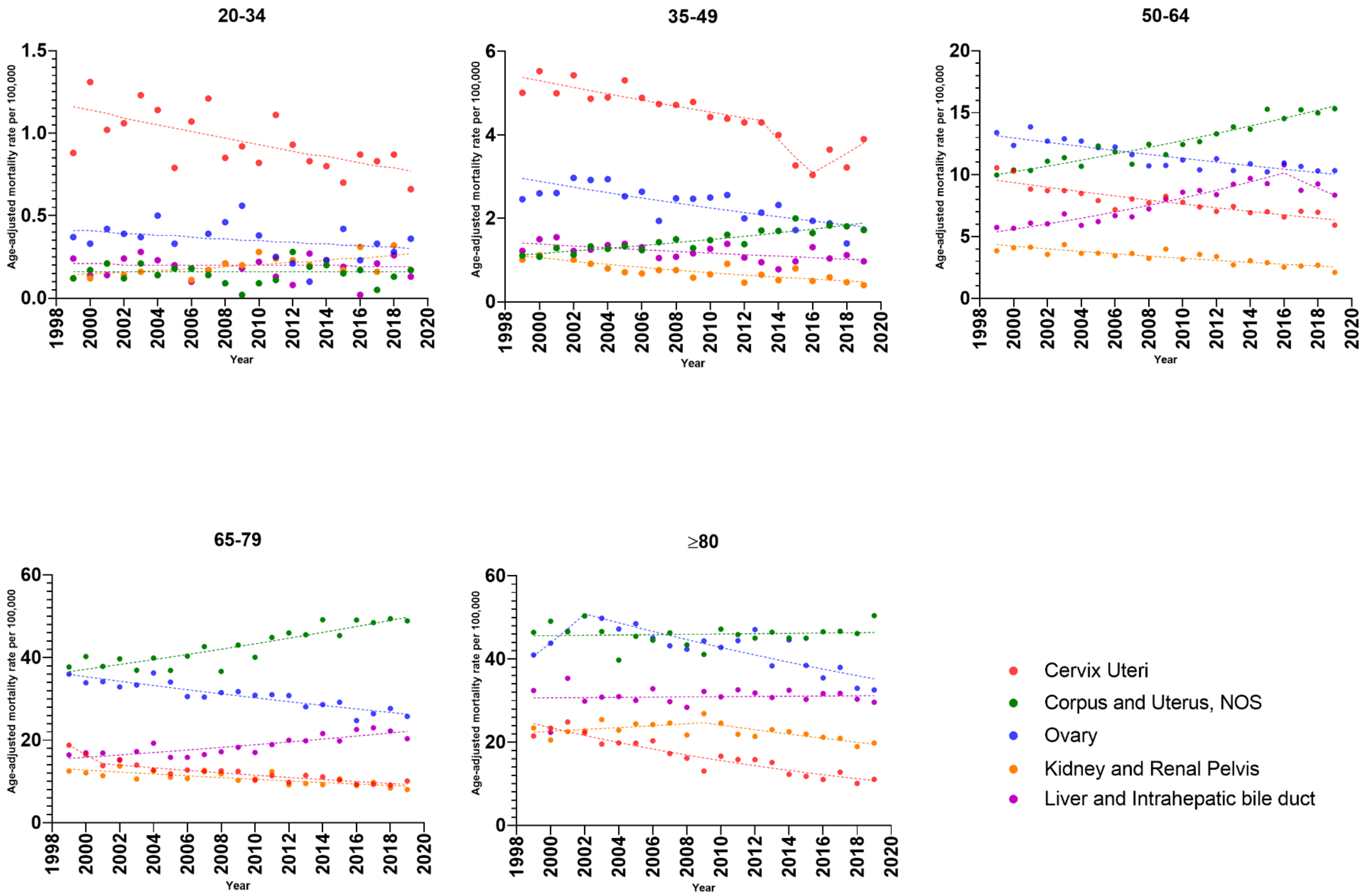

(Continued) eFigure 3. Trends in age-standardized death rates (1999-2019) among Black women by cancer site and age group. Death rates covered the entire US population. Trends were estimated using joinpoint regression and characterized by rate per 100,000.
Note: "---"represents modeled age-adjusted and "●" represents observed age-adjusted rates.

© 2022 Lawrence WR et al. *JAMA Oncology.*

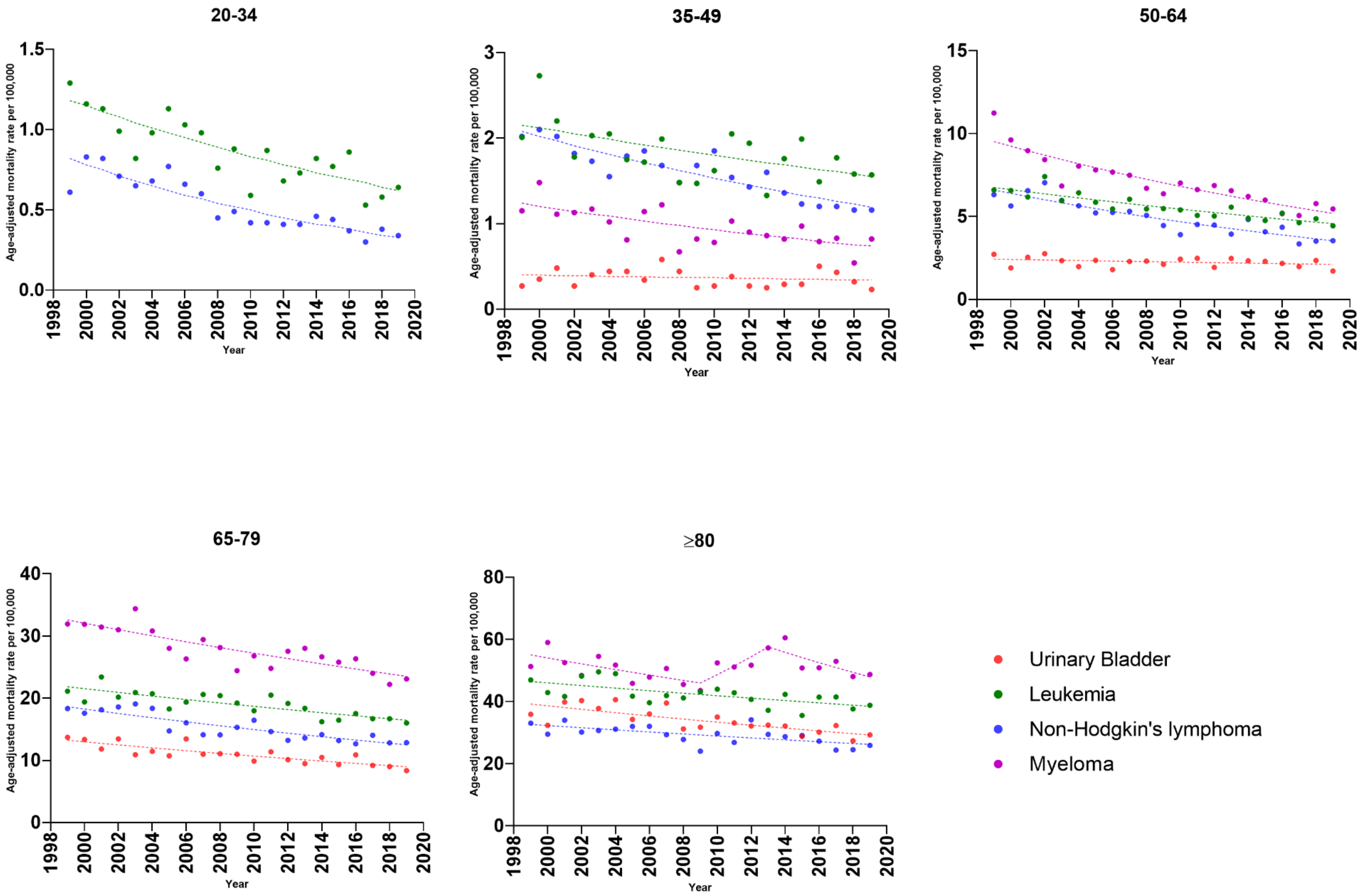

(Continued) eFigure 3. Trends in age-standardized death rates (1999-2019) among Black women by cancer site and age group. Death rates covered the entire US population. Trends were estimated using joinpoint regression and characterized by rate per 100,000.
Note: “---“represents modeled age-adjusted and “●” represents observed age-adjusted rates.

© 2022 Lawrence WR et al. *JAMA Oncology.*
